# The Relationship Between Manual Dexterity and Toothbrushing Efficiency in Preschool Children: A Crossover Study

**DOI:** 10.3390/children11121498

**Published:** 2024-12-08

**Authors:** Lisbeth Macote-Orosco, Andrea Martín-Vacas, Marta M. Paz-Cortés, María Rosa Mourelle Martínez, M. Joaquín de Nova

**Affiliations:** 1 Faculty of Dentistry, Alfonso X El Sabio University, Villanueva de la Cañada, 28691 Madrid, Spain; lmacooro@uax.es (L.M.-O.); amartvac@uax.es (A.M.-V.); 2 Postgraduate Specialization Program in Paediatric Dentistry, Faculty of Dentistry, Complutense University of Madrid, 28040 Madrid, Spain; mrmourel@ucm.es (M.R.M.M.); denova@ucm.es (M.J.d.N.); 3 Department of Dental Clinical Specialties, Faculty of Dentistry, Complutense University of Madrid, 28040 Madrid, Spain

**Keywords:** paediatric dentistry, oral hygiene, toothbrushing, preventive dentistry, oral health

## Abstract

Aim: The aim of this study was to evaluate the relationship between oral hygiene (OH) efficiency and manual dexterity skills in Spanish five-year-old children using two fine motor tests. Methods: A cross-sectional study with a pre–post evaluation was designed. The children’s OH was measured according to the Silness and Löe plaque index (PI) before toothbrushing upon arrival at school (pre) and after supervised toothbrushing (post). Motor skills (children’s dexterity) were determined with the Visual Motor Skills and Resistance to Fatigue test (VMSRF test) and the scale for the Behavioural Assessment of Preschool Skills (BAPS scale). Data were analysed with the SPSS^®^ statistics software with a 95% confidence interval and bilateral significance. Results: One hundred and twenty-nine children were evaluated. The mean PI was 2.5, indicating generally poor OH in the sample. No difference in OH was observed between genders or in the brushing frequency categories. No significant differences in OH or the PI were found between right- and left-handed children either (*p* > 0.05). Significant differences were found between the OH category pre and post toothbrushing in the total sample, as well as in the various sextants and surfaces evaluated. The results of the VMSRF and BAPS tests indicate moderate visuomotor skills and fatigue resistance and a poor average skill level. A statistically significant improvement in the PI was found in children with higher scores in the VMSRF and BAPS tests. Conclusions: A significantly higher OH efficacy was found in children with better motor skills, although five-year-old children showed moderate visuomotor skills and fatigue resistance and a poor average preschool skill level.

## 1. Introduction

Bacterial plaque directly influences the development of the most common oral cavity diseases, dental caries and periodontal disease [1,2,3,4,5]. Therefore, controlling dental plaque is essential for preventing these conditions. The most common and well-known practice is toothbrushing [6,7,8,9], and it is crucial to set up this habit at an early age [9]. Brushing teeth at home, twice a day, with parental supervision, has proven to be highly effective in preventing dental caries in children [10,11,12,13].

Multiple factors influence the effectiveness of toothbrushing in children [14], including the techniques used [15], frequency [16], socioeconomic status [17,18,19,20,21,22,23,24,25,26], area to be cleaned, applied pressure, duration [8], motivation [27,28,29,30], and manual dexterity [31], as well as individual characteristics [32,33,34,35,36,37]. However, multiple factors related to the children (development or independence), the families (hygiene habits, role models, knowledge, and attitudes), or the community (opportunities) influence OH behaviours [38].

The effect of OH education techniques on children has been studied over time. In the literature, we find various studies, including some that evaluate the effectiveness of two types of toothbrushes [39], toothbrushing performance after health education [40,41,42], differences in toothbrushing learning in the reduction in plaque and gingival inflammation [43], toothbrushing effectiveness in children and parents [44], effect of toothbrush grip [45,46], influence of OH practices [38], toothbrushing skills [47], toothbrushing duration and parental factors [48], OH habits and self-regulation skills [49], and toothbrushing patterns and behaviour [50,51,52].

Brushing teeth requires complex manual motor skills, which are not only age-dependent, making it challenging for very young children to master [53,54,55], but also influenced by individual dexterity and motivation [56]. Two-year-old children often show an interest in brushing their teeth independently; however, it is essential to recognize that their fine motor skills are still underdeveloped, necessitating parental supervision [10,57,58,59,60,61]. According to several authors, six-year-olds can progressively take on responsibility for their dental hygiene, albeit with adult supervision [9,10,45,53,61,62]. Some researchers suggest that by age eight, children can brush independently [14,63,64], although Sandström et al. [54] argue that aid may be needed until age ten. Casals-Peidró [65], in a study on oral hygiene (OH) habits in the Spanish school-age and adult populations, found that only 42.1% of parents aid in brushing their children’s teeth under the age of six. The study noted that parental involvement plays a role in achieving and maintaining a desired level of oral health in children [66]. A study published in 2021 elaborated on a novel tool to assess the basic activities of daily living in Spanish preschoolers, including toothbrushing as an important personal hygiene milestone in five-year-old typical children. No differences were found by gender, although significant differences were found between 3–4- and 5–6-year-old children, with better brushing efficacy in the older group [67].

Manual dexterity is the ability to perform tasks requiring precision and coordination in hand and finger movements. This skill is essential for activities that demand fine control, speed, and accuracy in the manipulation of objects or tools. Manual dexterity entails a combination of both gross motor skills (GMSs) and fine motor skills (FMSs). FMSs involve small and precise movements, while GMSs include broader manual abilities. Factors such hand–eye coordination, muscle strength, practice or experience, and neurological conditions influence manual dexterity. The neurological development of a child occurs gradually through various phases, helping them acquire new skills which include GMSs and FMSs. These comprise the control and coordination of the muscles of the upper extremities and are related to the functioning of the central nervous system, peripheral nerves, skeletal muscles, or joints [68,69,70]. Fundamental skills in children are influenced by several factors, such as the children’s age, sex, BMI, physical activity, sleep duration, perceived motor competence, and physical fitness [71,72]. While physical activity is related to an increase in fundamental motor skills, overweight is negatively associated with the development of motor skills [73]. The first five years of life are strongly associated with the emergence of a wide range of GMSs and FMSs. Toothbrushing is considered an FMS, as it requires precision and an elevated level of coordination [57,58]. The skills involved in fine motor movements develop over time, through experience and knowledge, and require normal intelligence and sensitivity, muscle strength and tone, and coordination [69].

There are various methods to determine the dexterity function for adults and children such as the Tyneside Pegboard Test, the Purdue Pegboard Test, the Minnesota Manual Dexterity Test (MMDT), the Minnesota Rate of Manipulation Test, the Archimedes spiral test (AST), the Jebsen Taylor Hand Function test, the Box and Block tests, and the Nine-Hole Pegboard Test. These tests are used in various healthcare sectors to determine the motor skills of the hand including the GMSs, FMSs, and grip strength [74]. The ABC test developed by Laurence Filho [75] and the scale for the Behavioural Assessment of Preschool Skills developed by Lozano and Sánchez [76] have also been used in Spanish-speaking countries in Latin America [77,78], but there are no studies conducted in Spain specifically analysing the relationship between manual dexterity skills and toothbrushing in children. Studies evaluating the relationship between manual skills and toothbrushing in preschoolers have been conducted in other countries such as Lithuania, Colombia, India, and Singapore [57,79,80,81]. However, due to cultural differences and varying family dynamics, it is essential to analyse this relationship within the Spanish population to assess the status of fine motor skills in preschoolers and its potential relevance in establishing preventive plans and OH education programmes. Enhancing knowledge across different cultures, races, and educational models broadens our understanding of psychomotor development, specifically manual dexterity, in children and its relationship with oral hygiene performance. Additionally, according to data from the 2020 Oral Health Survey in Spain, 35.5% of children aged 5–6 years old have already experienced dental caries, with 28.3% presenting active caries [82]. This highlights the importance of establishing a national OH education plan, according to psychomotor development and manual dexterity, to prevent and detect dental caries in preschool children. Therefore, the aim was to evaluate the relationship between OH efficiency and manual dexterity skills in Spanish five-year-old children using two fine motor tests.

## 2. Materials and Methods

### 2.1. Study Design

A multicentric, non-randomized, observational, descriptive, and analytic study was conducted. The study design included a cross-sectional and longitudinal evaluation of the study variables (pre–post), and this manuscript was prepared according to the Reporting stAndards for research in PedIatric Dentistry (RAPID) [83]. The research team was composed of paediatric dentists with wide clinical expertise previously calibrated.

### 2.2. Ethics Statement

This research was conducted with the approval of the ethics committee of the Clínico San Carlos Hospital (code 16/342-E, 4 August 2016) and complies with the requirements of the Declaration of Helsinki for biomedical research. Parents or legal guardians were properly informed with a written informational document, and Informed Consent was obtained prior to this study. The children’s assent was also required to participate in this study. Due to national and European data protection regulations, all participants were encoded and blinded both in the data sheets and database. The principal investigator administered the data during this study and eliminated personal data after this research was finished.

### 2.3. Participants and Sample Size

Several schools located in the Community of Madrid were invited to participate in this study, although only three schools accepted to take part, CEIP San Juan Bautista public school (28043, Madrid, Spain), Madres Concepcionistas School (28008, Madrid, Spain), and CEIP Príncipe de Asturias School public school (28049, Madrid, Spain). The average income per person was EUR 15,048, EUR 22,152, and EUR 18,573 in the districts of Ciudad Lineal (CEIP San Juan Bautista, Madrid, Spain), Moncloa/Aravaca (Colegio Madres Concepcionistas, Madrid, Spain), and Fuencarral/El Pardo (CEIP Príncipe de Asturias), respectively. All districts fall under the middle-class social category based on the per capita income classification for the city of Madrid, which has an average income per person of EUR 15,717. Due to all schools having the same socioeconomic status (middle class), the sample was not stratified by social class. During the preliminary visit to the schools, the available resources were assessed, revealing that none of them had OH facilities, medical services, or nursing staff. Therefore, no distinction was made between public and semi-private/private schools.

Due to the heterogeneity in previous studies, the sample size calculation was carried out in theoretical terms and not with previous data. The sample size was calculated using G*Power software (version 3.1.9.7., Düsseldorf, Germany) with the a priori procedure for differences between two dependent means (repeated measures, within factors) [84,85]. Eighty subjects were needed with an alpha error (p) of 0.05, 95% power, and an effect size as in SPSS (0.412). To solve a possible loss of 50% of the subjects due to cooperation, the initial sample size was increased to 120 subjects. The inclusion criteria were (1) five-year-old children, (2) without a psychomotor alteration diagnosis and (3) whose parents or legal guardians signed the Informed Consent. Children who did not cooperate in the oral examination or with systemic diseases implying psychomotor alterations were excluded from this study. The total sum of all five-year-old children in the three studied schools was 140; due to this, all children in the age range were selected. The age was chosen according to the previous literature, as hand dominance or preference is established at the age of four, and the first five years of life are strongly related to the development of a wide range of GMSs and FMSs.

### 2.4. Outcomes

Demographic and filiation data (gender, age, civil status, and educational level) were obtained from parents or caregivers with a questionnaire. Parental OH habits and the children’s toothbrushing practices were collected with a questionnaire and categorized as proper or improper regarding both the parents’ OH and that of their children. Parents and caregivers were asked about their perception of their oral health status (very good, good, moderate, and poor).

**Figure 1:**
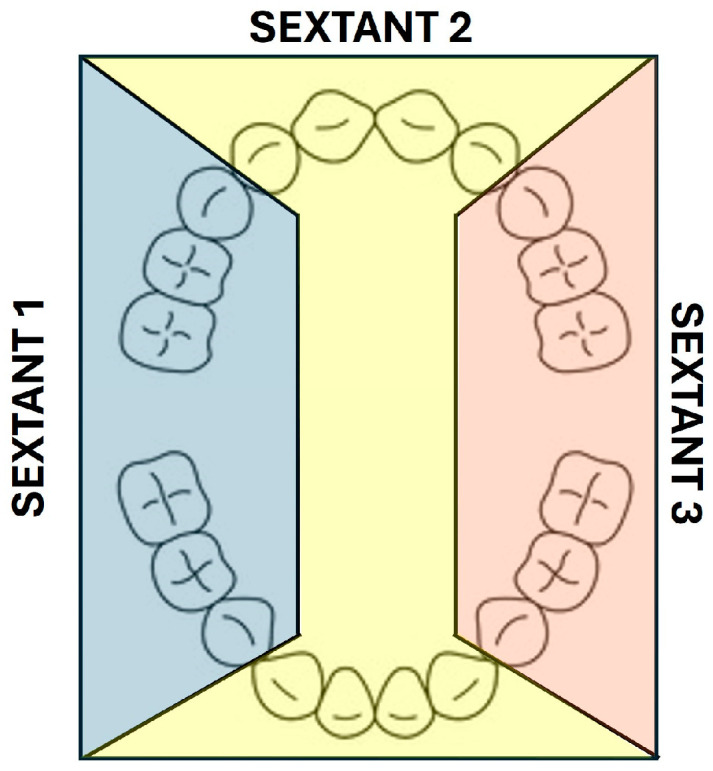
Teeth included in each evaluated sextant.

The children’s OH was measured according to the Silness and Löe plaque index (PI) [86,87] analysed with dental plaque disclosing tablets (Hagen Werken^®^, Hager & Werken GmbH & Co. KG, Duisburg, Germany), coded as (1) no dental plaque in the gingival area, (2) a thin film of dental plaque on the free gingival margin and the adjacent area of the tooth detected by passing a probe or using a disclosing agent, including chromogenic staining, (3) the moderate accumulation of soft deposits within the gingival sulcus, on the margin, and/or adjacent to the tooth surface, clinically visible, and (4) abundant soft material, with a thickness of 1–2 mm, extending from the gingival pocket and/or over the gingival margin and adjacent tooth surface. The PI was calculated as the sum of the numeric values of gingival units divided by the number of dental units. The scores from the four areas of each tooth were summed and divided into four to determine the tooth’s PI. The scores of individual tooth groups were grouped into sextants (Figure 1) to designate the PI for tooth groups: sextant 1 (right maxillary and mandibular molars and canines), sextant 2 (maxillary and mandibular incisors), and sextant 3 (left maxillary and mandibular molars and canines). The PI of the buccal and lingual surfaces was also calculated. The patient’s PI was obtained by summing the individual tooth indices and dividing by the number of examined teeth, and OH was categorized as good (PI = 0–0.5), moderate (PI = 0.6–1.5), or poor (PI = 1.6–3). The PI was measured before toothbrushing upon arrival at school (pre) and after supervised toothbrushing (post), calculating the variable PI improvement to assess the efficiency of autonomous brushing.

Motor skills (child’s dexterity) were determined with two fine motor tests adapted to 5-year-old children, the Visual Motor Skills and Resistance to Fatigue test (VMSRF test) and the scale for the Behavioural Assessment of Preschool Skills (BAPS scale). The selection of motor skills tests was based on their validation in Spanish with preschool-aged children and their ease of use. Both tests were supervised by the classroom teachers. The VMSRF test is a subtest of the ABC psychopedagogical test developed by Laurence Filho [75,88,89]. Item 8 of the ABC test evaluates attention and fatigue in children (VMSRF) and consists of performing well-defined tasks with dots in a 1 cm × 1 cm square as quickly as possible and in a specific order for 30 s individually. Following the rules of assessment of the test, four categories were established: very low (0–9 points), low (10–25 points), medium (26–50 points), or high (>50 points). The BAPS scale [76] consists of cutting out with scissors, without a time limit, the shape of a figure (a dove was chosen) drawn on a sheet of paper. The results of the test were divided into 4 categories: very bad (leaving more than half a centimetre between the shape and the dotted line or cutting into the shape more than half a centimetre), bad (although the cut may be irregular, the silhouette of the cut-out is unequivocally reminiscent of the shape), regular (the cut follows the dotted line of the silhouette, but has some less well-kept parts), or good (execution perfectly adjusted to the shape).

### 2.5. Study Procedure

The study procedure was conducted between September and June, and data analysis was conducted between July and September 2024. Firstly, the researchers and the School Principals arranged a reunion to coordinate the study phases and prepare the Informed Consent according to each school’s norms. Before the start of this study, Informed Consent and parental questionnaires were collected alphabetically, and a brief study explanation was conducted for all children in the classroom. Prior to the start of this study, all participants (teachers and dentists) underwent calibration. All procedures were carried out in groups, with children divided by classrooms, containing a maximum of 10 children per classroom due to logistic reasons.

Motor skill tests were conducted by the schoolteachers, blinding the results of the tests until data analysis. The proper execution of the tests was confirmed, and the hand dominance or preference (right or left) was registered. The hand dominance was provided by teachers through the daily observation of the children. The teachers at the schools were informed during the preliminary visit prior to the study day and were trained in the assessment of psychomotor tests. Due to logistical reasons, the psychomotor tests were administered by the classroom teachers of the evaluated groups, who remained uninvolved in the assessment process and exclusively supervised the procedure. The evaluation of the tests and the categorization of results were conducted in a blinded manner by other operators (M.R.M.-M. and M.J.d.N.), without knowledge of the PI results.

All oral explorations were conducted by the same paediatric dentist (L.M.-O.), previously calibrated with the other researchers, to prevent examination bias. The participating dentists are highly experienced paediatric dentists affiliated with the Department of Clinical Dental Specialties at the Complutense University of Madrid (UCM). The researchers are university professors, including the Director of the Postgraduate Specialization Programme in Paediatric Dentistry (M.J.d.N) at UCM.

The calibration process for OH assessment involved reviewing intraoral photographic series and evaluating the PI until all participants achieved consistent results. Consequently, the operator (L.M.-O.) was calibrated with all of them. Initial oral explorations (pre) were conducted according to WHO guidelines [90] in the classroom next to a window, with natural light between 9 and 13 am, with only a dental mirror after the use of a plaque disclosing tablet (Hagen Werken^®^, Hager & Werken GmbH & Co. KG, Germany). Immediately after, an informative and educational session on toothbrushing techniques, including toothbrush grip, toothbrushing importance, and motivation, was conducted. The OH education sessions were conducted through the presentation of audiovisual material (videos) in the classrooms, followed by brushing training on a dental model led by the paediatric dentists and the children.

After snack time and recess, according to international public health recommendations, an extra-soft paediatric toothbrush (Colgate^®^) and a 1000 ppm fluoride toothpaste (Colgate^®^) was provided to each child, and they were asked to brush their teeth for a controlled period of two minutes. Extra-soft toothbrushes were selected due to the sample age to prevent gum injuries. Toothbrushing was supervised without intervention by the researchers. After brushing, and after spitting the toothpaste in a plastic cup, they were given a plaque disclosing tablet (Hagen Werken^®^, Hager & Werken GmbH & Co. KG, Germany). Immediately after, a second dental exploration (post) was conducted in the same conditions as the pre-exploration to estimate the amount of plaque present on the different surfaces and locations. After dental exploration, parents or caregivers were informed individually about their children’s main oral findings and OH status.

### 2.6. Blinding

The principal investigator was blinded to the two motor skills tests used during oral evaluation in order to prevent assessing biases. Also, results of the pre-test were blinded to the principal investigator during the post-test. In addition, as previously established, the operators who analysed and categorized the psychomotor tests were external operators (not the schoolteachers), blinded to the plaque index results obtained.

### 2.7. Statistical Methods

Data were collected in an Excel^®^ spreadsheet (Microsoft, Inc. Realmond, Washington, DC, USA) and analysed with the statistical program IBM SPSS^®^ statistics software, version 24 (IBM Armonk, NY: IBM Corp.). Descriptive statistics were obtained (frequency, percentages, mean, and standard deviation), and the adaptation to a normal distribution was evaluated with the Kolmogorov–Smirnov test. Differences in OH were studied by applying the Chi-square (χ^2^) and U Mann–Whitney tests. Correlation between motor skill tests and OH was analysed with the Pearson correlation coefficient and Kendall’s Tau-b test. Kendall’s Tau-b analysis was used to examine ordinal relationships, and the Pearson correlation was used for continuous variables. Values of *p* < 0.05 (95% confidence interval) were considered statistically significant, applying an asymptotic or bilateral significance.

## 3. Results

### 3.1. Study Sample Characteristics

Although the initial sample included 140 children, a total of 129 preschool children (55 boys and 74 girls) were evaluated, as 11 children could not be assessed due to a lack of cooperation. The normality test results revealed that the PI did not follow a normal distribution (Kolmogorov–Smirnov *p* < 0.001 in all comparisons).

### 3.2. Evaluation of Parental Oral Hygiene Habits and Oral Health Status

A total of 75.8% of surveyed parents were categorized as having adequate OH habits; however, only 64.3% of parents reported regular dental visits, and 49.6% brushed their teeth three times a day as a routine. Additionally, only 59.7% of the surveyed parents and caregivers reported brushing their children’s teeth daily (only 7% three times per day and 48.7% twice a day), indicating that most parents fell into the group with inadequate (poor) OH care for their children. Most surveyed parents or caregivers reported their oral health status as either good (52.7%), very good (16.3%), or excellent (3.1%), while 18.6% and 1.6% of respondents indicated a fair or bad oral health status, respectively.

The relationship between parents’ habits and the OH care of their children was analysed, revealing that 72% (*n* = 18) of parents with inadequate OH habits also inadequately cared for their children’s OH, while 45.1% (*n* = 37) of parents with good OH practices also ensured proper care for their children. However, no statistically significant differences were found (χ^2^ *p* = 0.128). Significant differences (χ^2^ *p* = 0.018) were identified between OH habits and self-perceived oral health: among parents with good OH habits, 23% rated their oral health as very good and 60.9% as good, whereas among those with poor habits, 44.8% rated it as good and 41.4% as fair.

### 3.3. Oral Hygiene Evaluation

The mean initial PI was 2.5 (range 2–3), indicating generally poor OH in the sample. The category distribution showed that no child exhibited good OH, with the sample ranging between fair and poor (Table 1). No differences in OH were observed according to gender or hand dominance, neither in the toothbrushing frequency categories (χ^2^ *p* = 0.334 and *p* = 0.364, respectively) nor in the PI outcome (U Mann–Whitney *p* = 0.336 and *p* = 0.238, respectively).

The post-brushing PI remained within the poor category (PI = 2.22 ± 0.437); however, in the categorical analysis, an increase in children with good OH (42.6%) was observed compared to the initial assessment (Table 1). The influence of gender on post-brushing OH was analysed, revealing that boys exhibited better OH than girls across all categories, with a significantly lower post-brushing PI compared to girls (U Mann–Whitney *p* = 0.012, mean difference = −0.26). The effect of dominant side on post-brushing hygiene was also analysed, with no significant differences found (χ^2^ *p* = 0.155, U Mann–Whitney *p* = 0.056).

In the sextant distribution, sextant 3 showed the best OH (mean PI = 2.35), while sextant 1 had the poorest (mean PI = 2.49) (Table 2). The potential influence of gender and dominant hand (right- or left-handed) on OH across different sextants was analysed, with no statistically significant differences found (U Mann–Whitney and χ^2^ *p* > 0.05 for all comparisons.

**Figure 2:**
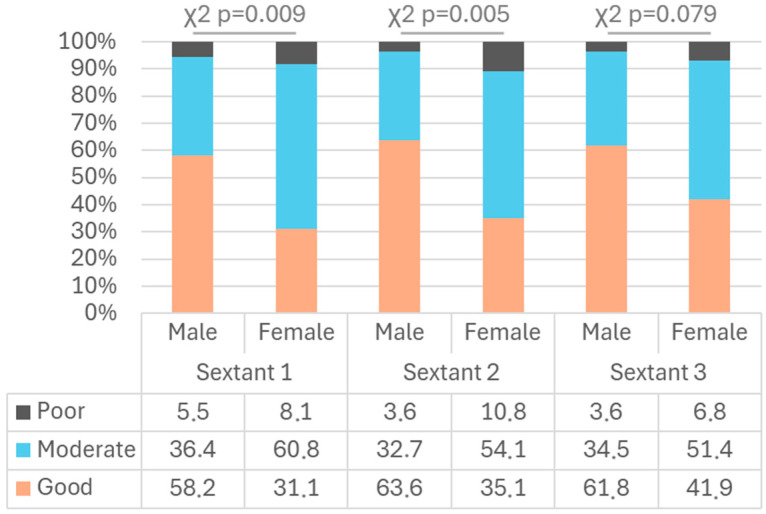
Evaluation of OH categories according to gender and sextant.

Post-brushing OH was evaluated by sextants, revealing that sextant 2 had the highest PI, followed by sextants 1 and 3 (Table 2). The PI was significantly lower in boys compared to girls across all sextants evaluated. The influence of gender on the post-brushing PI was analysed (Figure 2), showing significant differences in sextants 1, 2, and 3 evaluating the PI (U Mann–Whitney *p* = 0.004, *p* = 0.01, and *p* = 0.026, respectively) and OH categories (χ^2^ *p* < 0.05 for all comparisons), with a lower post-brushing PI in boys than in girls in sextants 1, 2, and 3 (mean difference = −0.30, −0.36, and −0.23, respectively). The effect of hand dominance on the post-brushing PI was also studied, with no significant differences found (U Mann–Whitney and χ^2^ *p* > 0.05 for all comparisons).

OH was analysed by tooth surfaces, finding that the buccal surface showed greater plaque accumulation than the lingual surface across all locations studied (Table 3). The influence of gender on the OH of the surfaces analysed was examined, revealing that overall, gender did not affect toothbrushing on either the buccal or lingual surfaces (U Mann–Whitney *p* > 0.05 for both comparisons). However, by sextant analysis, the buccal surface in sextant 1 showed a significantly lower PI in boys than in girls (U Mann–Whitney *p* = 0.032, mean difference = −0.189), with no significant differences on other surfaces (U Mann–Whitney *p* > 0.05 for all comparisons). Additionally, the PI differences between right- and left-handed children were evaluated, showing that right-handed children had a significantly lower PI on the lingual surface of sextant 1 compared to left-handed children (U Mann–Whitney *p* = 0.003, mean difference = −0.380), with no significant differences in the other evaluated variables (*T* test *p* > 0.05 for all comparisons). No differences were found in the OH categories by gender or hand dominance (left- or right-handed) on the surfaces evaluated (χ^2^ *p* > 0.05 for all comparisons).

**Figure 3:**
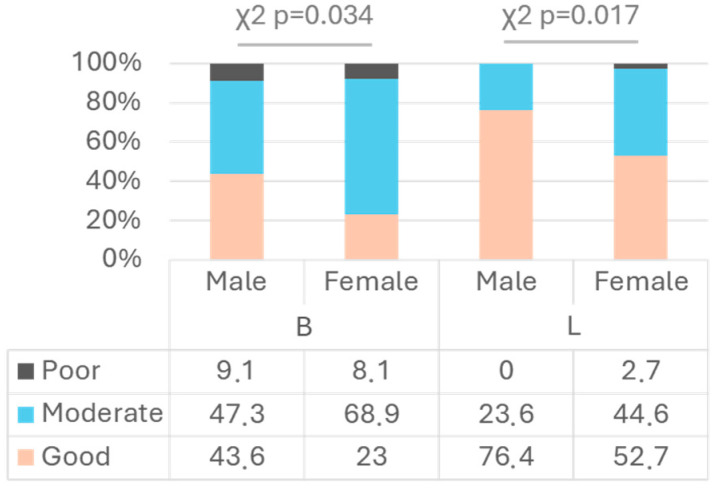
Evaluation of OH categories according to gender and buccal (B) or lingual (L) dental surfaces.

The PI and categories of post-brushing OH were analysed by tooth surface (Table 3), once again showing greater bacterial plaque accumulation on buccal surfaces than on lingual surfaces. When examining the influence of gender (Table 3), significant differences were observed on the buccal surface (U Mann–Whitney *p* = 0.042, mean difference = −0.20) and lingual surface (U Mann–Whitney *p* = 0.005, mean difference = −0.26), with a lower PI in the group of boys. Analysing the evaluated locations, a significantly lower PI was found on the buccal (U Mann–Whitney *p* = 0.003, mean difference = −0.33) and lingual surfaces (U Mann–Whitney *p* = 0.002, mean difference = −0.32) of sextant 2 in boys, as well as on the lingual surfaces in general (*T* test *p* = 0.004, mean difference = −0.26). No significant differences were found in the other evaluated locations (U Mann–Whitney *p* > 0.05 for all comparisons). In the evaluation of the OH categories (Figure 3), significant differences were found on the buccal (χ^2^ *p* = 0.034) and lingual surfaces (χ^2^ *p* = 0.017), indicating better OH in boys. The influence of hand dominance (right- or left-handed) on surface hygiene was analysed, with no statistically significant differences found (χ^2^ and U Mann–Whitney *p* > 0.05 for all comparisons).

The statistical analysis confirmed differences in the OH category pre and post toothbrushing in the total sample, as well as in the various sextants and surfaces evaluated (Kendall’s Tau-b *p* < 0.001 for all comparisons) (Table 4). These differences correspond to an improvement in hygiene categories. Among the children who initially exhibited moderate hygiene, 60.94% demonstrated good hygiene after brushing. Of the children who initially had poor hygiene, 24.62% achieved good hygiene post brushing, while 67.69% maintained moderate hygiene.

### 3.4. Evaluation of Fine Motor Skills in Study Sample

**Figure 4:**
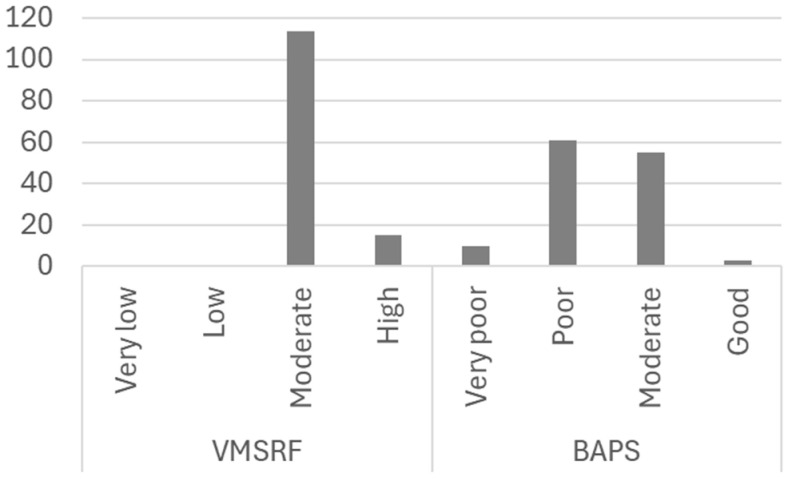
Motor skill evaluation according to VMSRF and BAPS tests.

Fine motor skills and preschool abilities were evaluated using the VMSRF and BAPS tests (Figure 4). The results of the VMSRF test indicate moderate visuomotor skills and fatigue resistance (2.12 ± 0.322), with scores falling into the average (88.4%) and high (11.6%) categories. Gender differences were analysed, revealing that while the differences were not statistically significant (χ^2^ *p* = 0.373), boys scored higher than girls. No differences were found regarding hand dominance (χ^2^ *p* = 0.129), although left-handed children performed better on the test. The BAPS test showed a poor average skill level (1.40 ± 0.666), with most children scoring poorly (47.3%) or moderately (42.6%). Although the differences were not statistically significant (χ^2^ *p* = 0.792), girls exhibited greater skills than boys. The analysis related to hand dominance showed that left-handed children were more skilled, although this difference was not statistically significant (χ^2^ *p* = 0.343). The relationship between the VMSRF and BAPS tests was analysed, finding no correlation between the tests (Pearson *p* = 0.392).

### 3.5. Correlation Between Fine Motor Skills and Oral Hygiene Post Toothbrushing

**Figure 5:**
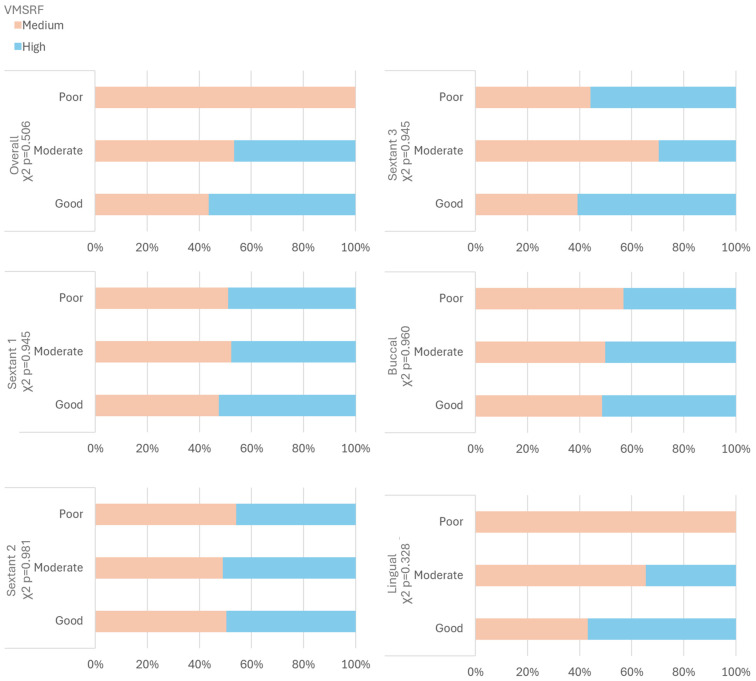
Analysis of VMSRF test and OH post brushing according to sextant and surface evaluation.

No significant correlation was found between the VMSRF test and the categories of post-brushing OH, both in the overall assessment (Pearson *p* = 0.506) and by sextants (Pearson *p* > 0.05 in all three sextants) and surfaces (Pearson *p* > 0.05 in all comparisons) (Figure 5). However, despite the lack of statistical significance, there is a trend suggesting better brushing among children with higher fine motor skills, particularly in total OH and on the lingual surfaces.

**Figure 6:**
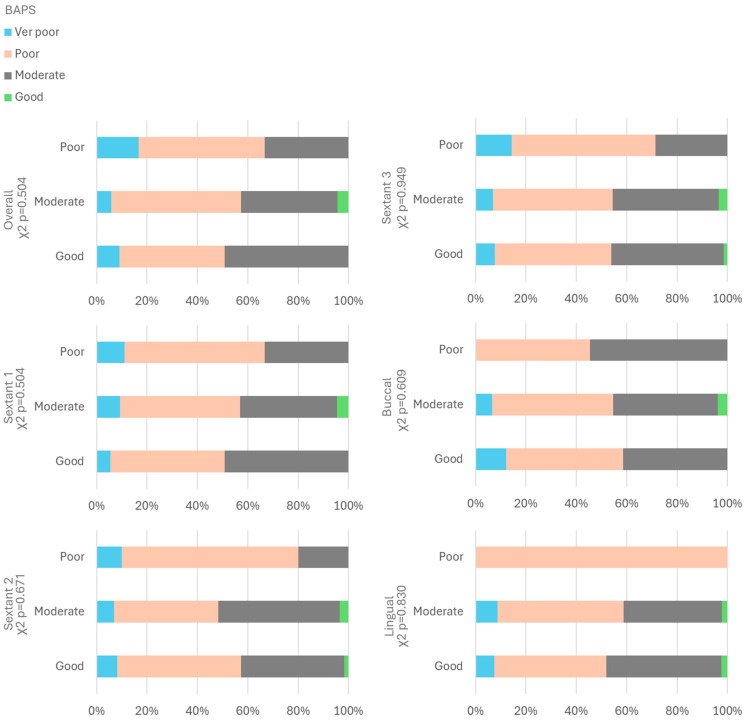
Analysis of BAPS test and OH post brushing according to sextant and surface evaluation.

No significant correlation was found between the BAPS test and the overall categories of post-brushing OH (Pearson *p* = 0.504), as well as by sextants (Pearson *p* > 0.05 in all three sextants) and surfaces (Pearson *p* > 0.05 in all comparisons) (Figure 6). Despite the absence of statistically significant differences, it can be observed that children with better skills tend to have improved OH.

### 3.6. Assessment of Improvement in Plaque Index According to Fine Motor Skills

The improvement in OH was assessed by calculating the difference between the pre- and post-brushing PI (Table 5), resulting in an overall improvement in the PI of 0.88 ± 0.608, with a greater improvement observed in the buccal surfaces compared to the lingual surfaces. Regarding the improvement by sextants, sextant 2 showed the greatest improvement in the PI, followed by sextant 1.

The influence of gender, hand dominance, and motor skills tests on the improvement in the PI was analysed. Regarding gender, although no differences were found in the overall improvement in the PI or across the evaluated surfaces, significant differences were observed in sextants 2 and 3 (U Mann–Whitney *p* = 0.015 and *p* = 0.032, respectively), with a more notable improvement in the group of boys compared to girls. In relation to the motor skills tests, differences were found in the overall PI, as the improvement in the PI was more pronounced in the group with higher scores on the VMSRF test (U Mann–Whitney *p* = 0.012). No differences were observed concerning hand dominance or the BAPS test in the change in the PI across the evaluated surfaces and locations (U Mann–Whitney *p* > 0.05 for all comparisons).

The correlation between the motor skills tests and improvement in the PI was analysed, revealing a positive and significant correlation between the VMSRF test score and the total PI (Kendall’s Tau-b *p* = 0.012). However, no significant correlations were found in the evaluation of the different sextants and surfaces (Kendall’s Tau-b *p* > 0.05 for all comparisons). The assessment of the BAPS test did not yield statistically significant differences for any of the comparisons (Kendall’s Tau-b *p* > 0.05 for all comparisons).

## 4. Discussion

### 4.1. Methods and Study Procedure

The study methodology was designed to analyse the relationship between FMSs and toothbrushing effectiveness in 129 five-year-old Spanish children of middle socioeconomic status. OH was assessed with the PI, the Silness and Löe index, in accordance with other authors [41,79]. In previous studies, several evaluation methods have been carried out, such as the Greene and Vermillion method [39], Turesky modification of the Quigley–Hein plaque index (TQHPI) [40,43,80], O’Leary method [50,91], Marginal Plaque Index [40], Oral Hygiene Index (OHI) [81,92], the simplified Debris Index (DI-S) [92], plaque control index (PCI) [49], papilla bleeding index (PBI), Nourullah and Splieth method [44], and Quigley–Hein Index (QH-I) [57]. Although the most common evaluation of brushing effectiveness is through the PI or brushing time, we found other evaluation methods such as the toothbrushing observation system (TBOS) [48], Perceived health Competence Scale [49], Treatment Self-Regulation Questionnaire [49], Self-Report Index of Habit Strength [49], innate or acquired skills in preschool children [42], and type of movement performed [57].

There are very few studies that evaluate the relationship between motor skills and toothbrushing [57,79,80,81]; we found studies with a heterogeneous sample size ranging from 42 to 941 children of different ages, and none of these studies were conducted in Spanish children. The age groups evaluated in previous studies are children aged 6–12 years old [80], 5–7 years old [81], 4–6 years old [79], or 3–9 years old [57]. These studies were conducted in countries such as Lithuania [79], Colombia [57], India [80], and Singapore [81]. FMS assessment has been conducted with the Archimedes spiral test [57,80] or the Five-To-Fifteen Revised (5-15R) questionnaire [57]. On the other hand, one study evaluated GMSs with the Manual Dexterity Test [80]. Motor development has also been studied with the Beery VMI test [81], including further movements/grip patterns or coordination [57]. In our case, we evaluated two FMS items using the VMSRF (ABC subtest) and BAPS tests, both of which are validated for the sample used. According to Vilca et al. [89], the ABC test consists of easily and quickly executable tasks. Montessori recommends that for the development of motor skills, the individual should perform preparatory exercises such as tracing and/or filling geometric shapes. The BAPS scale, as described by Rodríguez Laínez et al. [93], is an unbiased, standardized, and validated instrument endorsed by three groups of experts. Moreover, the reliability or internal consistency of the scale, measured by Cronbach’s alpha, exceeds 0.80. Activities such as cutting out shapes involve controlled and deliberate movements requiring significant precision and FMSs and play a crucial role in the subsequent development of manual dexterity.

### 4.2. Oral Hygiene Habits

There is strong evidence recommending that parents brush at least twice a day [94], in agreement with studies that evaluated the frequency of brushing in children by parents [23,38,39]. However, in another study, it was reported that only 24% of children performed toothbrushing twice a day [95]. Only 65–76% of parents of children under 3 years old acknowledged brushing their children’s teeth daily [96], decreasing to 28% in children aged 3–5 years old [97]. A study conducted in Spanish children determined that 61.3% of children aged 3–6 years old brushed their teeth at night, finding that girls brushed their teeth more frequently than boys [98]. Our results indicated that 48.7% and 7% of parents reported brushing their children’s teeth two or three times a day, respectively. Notably, 78.8% of parents stated that the children themselves were responsible for brushing, not them, with 98.6% of parents believing their children had sufficient skills to brush their own teeth.

Among the factors influencing OH behaviours, environmental factors (such as demands on attention with multiple children, social support), parental knowledge levels, and the development of parental skills in controlling and managing behaviour or in establishing and maintaining routines are highlighted [97,99]. Shaghaghian et al. [92] established that parental concern for their children’s dental health and OH is significantly associated with a high educational level of the parents, employed mothers and fathers, and the absence of prior visits to a paediatric dentist. Regarding the degree of parental knowledge about their children’s oral health, it is estimated that approximately 78–90% possess good knowledge [100,101], and a low level of knowledge is associated with poor OH behaviour in their children [102]. Vanagas et al. [103] analysed parental skills and attitudes related to the importance of developing proper OH habits in their children. It was found that only 52.6% of parents used an appropriate toothbrush, 69.7% brushed their children’s teeth two or more times a day, and 77.7% provided sugary snacks. Additionally, significant differences were observed between parental OH habits and their understanding of the importance of brushing, oral health, and their children’s nutrition. Similarly, our results reveal a trend suggesting that parents’ OH habits influence their children’s oral healthcare, although the differences were not significant.

Regarding socioeconomic status, there is clear evidence of its influence on dental status and OH. According to 2020 data from Spain, significant demographic differences were observed in the oral health status of 5- to 6-year-old children, with a lower incidence of caries among children from higher socioeconomic backgrounds [82]. A study conducted on Spanish children found that the frequency of dental visits and toothbrushing was positively correlated with the child’s age, parental educational level, and monthly household income [98]. In our case, all the children belonged to a middle socioeconomic level, so demographic differences were not analysed.

### 4.3. Oral Hygiene Educational Programmes

Regarding OH in young children, a study conducted in the 1980s [42] analysed 420 children between 2 and 4 years old, finding that only 86.4% were initially able to put toothpaste on the brush; however, all of them were able to bring the brush to their mouths. According to Benadof et al. [104], the development of oral brushing skills requires four different stages, and the transition between stages is not only age-dependent but is also significantly influenced by the parents’ knowledge of OH and their perception of their children’s physical, cognitive, and motor development skills. These stages are initiation and completely dependent toothbrushing, assisted toothbrushing, the transition to independent toothbrushing, and independent toothbrushing.

Children who participate in oral health education programmes have been demonstrated to have reduced dental plaque [49]. In a systematic review conducted in 2015 [105], the authors stated that OH education improves both plaque and gingival indices, especially when it involves demonstration and supervision. However, the evaluation of the results obtained in all cases is short term, as in the present research, so we can conclude that while knowledge increases, attitudes and behaviours do not increase proportionally. On the other hand, another systematic review [106] stated that there is insufficient evidence regarding the effectiveness of home OH programmes due to the lack of scientific rigor in the studies.

### 4.4. Oral Hygiene in Children

No data regarding the PI in five-year-old children were found in the literature, but data are available for older age groups. In a study conducted where different OH education methods were evaluated in 12-year-old children, pre-brushing PI values ranging from 1.33 to 1.47 were found, with a significant improvement after brushing (post-brushing PI = 0.62–1.01) across all education methods [41]. In another study with children aged 8–12 years old, significant differences were also found between the pre-treatment PI (1.64), immediately after treatment (0.81), and at seven days (0.81) following OH education [45]. Our pre-brushing PI results were higher than those reported in older children (PI = 2.5), showing only a slight decrease after toothbrushing (PI = 2.2). According to our results, studies evaluating different types of toothbrushing learning found that in all cases, brushing improved [40,107], and the improvement was greater in cases where education included instruction and demonstration, with a more significant improvement in the posterior sectors compared to the anterior region [43]. Regarding the OH categories, a study conducted on children aged 5–7 years old reported that before brushing, 91% of the children had poor OH, while after brushing, 58.6% of the children had moderate hygiene and 41.4% had poor hygiene, showing an improvement in the OH category in 49.5% of cases [81]. Our results are consistent, as prior to brushing, the children were categorized into the moderate (50.4%) and poor (49.6%) OH categories. However, in the post-brushing evaluation, 42.6% of the children were in the good OH category, 52.7% were in the moderate category, and the remainder were in the poor category.

The effect of age and gender has been widely studied, concluding that chronological age shows a direct correlation with brushing effectiveness. Regarding gender, many studies found no significant differences in OH between boys and girls, but there is a trend toward better brushing in girls compared to boys [7,8,21,32,102,108,109,110]. Our data reveal the absence of differences in pre-brushing OH by gender but a better PI post brushing in boys. However, when evaluating the difference in the PI before and after brushing, no differences were found, consistent with the previous literature.

It has been observed that electric toothbrushes are more effective in reducing the PI in young children [111,112,113], despite authors such as Silverman et al. and Chua et al. [81,114] stating that the differences are not clinically significant. Additionally, three-sided brushes to be more effective than manual brushes [39]. Regarding toothbrush materials, there are no differences between nylon and silicone bristle toothbrushes. In relation to the type of toothbrush grip, Pujar et al. [91] stated that although the grip influences the effectiveness of PI reduction, the differences are not statistically significant. Additionally, the authors stated that the distal oblique grip is the most common among children, in agreement with Sharma et al. [45]. However, in other studies [46,47], it was found that the most frequently used grip type was the distal grip (73%), followed by the power grip (43%) and the oblique grip (29%), with no differences between age groups. In other studies, a palm grip was observed to be more frequent [50,52]. In relation to the type of movement in a study evaluating 12-year-old children [40], the authors establish that the preferred brushing movement for the buccal surfaces is circular movements, and for the inner surfaces, it is vertical movements, followed by horizontal movements in both cases, in agreement with other studies [51]. Furthermore, the authors established that the duration of circular movements and the number of sextants brushed are predictors of PI improvement. In other studies, the authors found that most children performed horizontal movements [50,52].

The duration of brushing and plaque accumulation are highly correlated [32,115,116]. Effective toothbrushing should be performed for a minimum of two minutes [30,117,118,119,120], although studies show that actual brushing time ranges between 28 and 47 s [46]. However, in another study, it is stated that the improvement in OH has a proportional relationship to age [91].

Regarding the sextants, it has been observed that children spend more time brushing the anterior sextants on their outer surfaces, perhaps due to easier access [8,11,51]. Due to their greater ease for right-handed patients, the left sextants also showed better OH [121]. Honkala et al. [8] stated that the anterior and left sextants achieve better brushing. In our results, the left sextant presented the best pre-brushing OH, while the right sextant had the worst, with no gender differences. Regarding the post-brushing evaluation after OH education, the anterior teeth showed the best brushing efficacy, followed by the right and left teeth. Significantly better brushing was observed in boys compared to girls across all sextants. Hand dominance did not influence the brushing in any of the comparisons. The improvement in the PI after brushing was most pronounced in the right posterior teeth, followed by the left posterior teeth, as the anterior teeth already had a lower pre-brushing PI. The buccal surfaces of the teeth presented a higher plaque accumulation before brushing compared to the lingual surfaces, which have greater self-cleaning capacity [54,55]. In our results, we also found worse OH in the buccal areas of the teeth, again observing better OH in the boys compared to the girls in certain regions. Additionally, our results showed a greater improvement in the OH performance on the buccal surfaces than on the lingual surfaces after OH education.

### 4.5. Relationship Between Motor Skills and Oral Hygiene Efficacy

Traditionally, manual dexterity has been considered a predictor of OH [34,42]; however, it seems that these differences are not significant following re-evaluation after 15 days [35]. In a study comparing toothbrushing skills in preschoolers between 2000 and 2010 [79], the data revealed a general trend towards a decrease in the percentage of children brushing their teeth regularly, with an increase in the percentage of irregular brushing among girls. However, a significant reduction in bacterial plaque accumulation was observed in 2010, which could be associated with better brushing skills and regular brushing with adult supervision. This longitudinal difference was only observed in boys. The results of the FMS tests conducted in our study indicate moderate–high visuomotor skills and fatigue resistance in five-year-old Spanish children, with no differences by gender or hand dominance. The skill level of our sample oscillates between the poor and moderate categories. Although no differences were found in either test by gender or hand dominance, there was a tendency for girls and those with left-hand dominance to have better results than boys.

In a study conducted with children aged 7–12 years old, the authors confirmed that as age increases, both FMSs and GMSs improve [80]. In a study with children of the same age, the authors state that, regardless of age, children with better FMS abilities show a greater mean difference in the PI before and after brushing, indicating better brushing efficacy [80]. In a recent study conducted with Spanish children of similar age to ours (3–6 years old), basic daily activities were analysed, evaluating toothbrushing within the OH category. The study concluded that manual dexterity significantly influenced the performance of toothbrushing [67]. Chua et al. [81] analysed motor development and OH in children aged 5–7 years old using a subjective scale (parental perception) and an objective one (VMA test). The authors found that children with better motor skills, such as writing or drawing, tying shoelaces, or cutting their nails, were more likely to improve their OH category. However, no differences were found in the objective evaluation of motor skills using the VMA test and toothbrushing efficacy. In a study conducted with children aged 3–9 years old [57], girls demonstrated a significantly more typical pattern than boys. Additionally, regarding the movements and grasp patterns related to FMSs, older children exhibited fewer problems than younger children during toothbrushing. Furthermore, in the gender analysis, it was found that boys exhibited more frequent hand, shoulder, and arm movements during the brush grip than girls. Variables such as age, gender, and complex rotation were significantly associated with brushing effectiveness, with age being the only variable showing a negative association. No differences were found between the genders. In line with the previous literature, our results indicated no differences in the PI or OH category post brushing depending on the FMS tests, although children with higher scores demonstrated better toothbrushing efficacy. However, when analysing the difference in the PI pre and post brushing, it was found that children with higher scores on the VMSRF test showed a more pronounced improvement in the PI, with no differences by sextants or surfaces.

### 4.6. Study Limitations and Strengths

A potential limitation of this study is the inclusion of preschool-aged children, who exhibit limited cooperation and attention. The presence of a single examiner, although previously calibrated, may introduce examiner bias, as it was not possible to conduct second measurements to analyse intra-operator agreement. Stratified sampling by subgroups of gender and hand dominance was not established, although the sample was balanced in terms of gender. However, the predominance of right-handed subjects in this study may result in unreliable outcomes for the left-handed population. Furthermore, children diagnosed with systemic disorders were excluded; however, those without a formal diagnosis but presenting motor difficulties were not screened and were therefore included in this study, which may have influenced the results obtained. The cross-sectional evaluation consisting of immediate pre- and post-assessments does not allow for an analysis of the long-term effects of OH education in preschool children or the persistence of the skills acquired during the informational session. The group assessment may have introduced procedural biases, such as distractions among the children; however, given that the groups were small (10 children), these biases are minimal. Additionally, factors such as the hardness of the toothbrush, the number of siblings or birth order, and the age at which brushing was initiated have not been analysed or discussed. For this reason, we believe it is necessary to apply the same methodology to older age groups in a long-term evaluation and compare the results to determine at what age children can be considered to have sufficient autonomy and manual skills to maintain adequate OH independently. Furthermore, school affiliation was not recorded, as all schools belonged to a middle socioeconomic class, making it impossible to determine the representation of each school. However, this may impact the generalizability of the data, as they may be less extrapolatable to other socioeconomic levels. The theoretical mathematical calculation of the sample size, due to the lack of published studies using the tests employed, may affect the robustness of the results. Nonetheless, the significance levels used are appropriate, ensuring that the data obtained, while requiring cautious interpretation, remain valid.

However, this is the only study conducted in Spain that directly examines the relationship between manual dexterity and toothbrushing efficacy in preschool children, using validated tools for motor skills assessment. Additionally, a key strength of this study lies in the inclusion of a large sample of preschool children from multiple schools, enhancing the representativeness of the results.

Recent studies have reported that the use of chatbot-type applications improves toothbrushing rates in children and caregivers, enhances perceptions of oral healthcare, and increases knowledge about oral health and toothbrushing techniques [96]. Other digital applications have also proven to be effective in promoting oral health behaviours in children [122]. Therefore, it would be valuable to incorporate these methods as part of OH education to assess learning outcomes and their retention through reinforcement sessions during follow-up visits. As a result, we propose the following clinical applications. We recommend systematic and repeated OH education for preschool children during dental check-ups, providing parents and/or caregivers with information on improvements in hygiene and diet, and assessing the children’s psychomotor development to adapt the brushing performance accordingly. Future investigations should be conducted evaluating the long-term results of OH education and other socioeconomic status and age categories.

## 5. Conclusions

A significant relationship was determined between the parents’ habits and the OH care of their children. Poor OH was found in Spanish five-year-old children, without differences by gender and hand dominance. The overall OH efficacy demonstrated no differences by gender or hand dominance. A significantly higher OH efficacy was found in buccal surfaces and anterior teeth, and boys exhibited a higher OH performance in anterior and left posterior teeth than girls. Preschool children exhibited moderate visuomotor skills and fatigue resistance and a poor average skill level. The OH efficacy was more pronounced in children with higher visuomotor scores, with no differences according to preschool skills. Manual dexterity and visuomotor skill evaluation should be incorporated in OH education planning.

## Figures and Tables

**Table 1:** Description of OH categories before (pre) and after (post) toothbrushing.

OH	TOTAL		Gender				Dominant Hand			
			Pre		Post		Pre		Post	
	Pre	Post	Male	Female	Male	Female	Right	Left	Right	Left
	N %	N %	N %	N %	N %	N %	N %	N %	N %	N %
Good	0 0%	55 42.6%	0 0%	0 0%	31 56.4%	24 32.4%	0 0%	0 0%	53 45.3%	2 16.7%
Moderate	65 50.4%	68 52.7%	30 54.5%	34 45.9%	22 40%	46 62.2%	60 51.3%	4 33.3%	59 50.4%	9 75%
Poor	64 49.6%	6 4.7%	25 45.5%	40 54.1%	2 3.6%	4 5.4%	57 48.7%	8 66.7%	25 4.3%	1 8.3%
TOTAL	129 100%	129 100%	55 100%	74 100%	55 100%	74 100%	117 100%	12 100%	117 100%	12 100%

**Table 2:** PI and OH pre and post toothbrushing.

Sextant	PLAQUE INDEX		OH					
	Pre	Post	Pre			Post		
	Mean ± SD	Mean ± SD	Good	Moderate	Poor	Good	Moderate	Poor
			N %	N %	N %	N %	N %	N %
Sextant 1	2.49 ± 0.517	2.16 ± 0.423	1 0.8%	64 49.6%	64 49.6%	55 42.6%	65 50.4%	9 7%
Sextant 2	2.47 ± 0.517	2.22 ± 0.455	1 0.8%	66 51.2%	62 48.1%	61 47.3%	58 45%	10 7.8%
Sextant 3	2.35 ± 0.517	2.12 ± 0.375	2 1.6%	80 62%	47 36.4%	65 50.4%	57 44.2%	7 5.4%

**Table 3:** PI and OH category by surfaces and sextants pre and post toothbrushing in the total sample.

Surface	PLAQUE INDEX		OH CATEGORY					
			Pre			Post		
	Pre	Post	Good	Moderate	Poor	Good	Moderate	Poor
	Mean ± SD	Mean ± SD	N %	N %	N %	N %	N %	N %
Buccal surface	2.72 ± 0.450	1.77 ± 0.593	0 0%	36 27.9%	93 72.1%	41 31.8%	77 59.7%	11 8.5%
Buccal surface—sextant 1	2.58 ± 0.495	1.77 ± 0.734	0 0%	54 41.9%	75 58.1%	53 41.1%	53 41.1%	23 17.8%
Buccal surface—sextant 2	2.70 ± 0.461	1.63 ± 0.638	1 2.9%	39 30.2%	90 69.8%	59 45.7%	59 45.7%	11 8.5%
Buccal surface—sextant 3	2.51 ± 0.502	1.60 ± 0.642	1 2.9%	63 48.8%	66 51.2%	62 48.1%	56 43.4%	11 8.5%
Lingual surface	2.22 ± 0.437	1.39 ± 0.520	1 0.8%	98 76%	30 23.3%	81 62.8%	46 35.7%	2 1.6%
Lingual surface—sextant 1	2.16 ± 0.423	1.32 ± 0.484	3 2.3%	103 79.8%	23 17.8%	89 69%	40 31%	7 5.4%
Lingual surface—sextant 2	2.22 ± 0.455	1.42 ± 0.596	2 1.6%	96 74.4%	31 24%	82 63.6%	40 31%	7 5.4%
Lingual surface—sextant 3	2.12 ± 0.375	1.29 ± 0.487	2 1.6%	109 84.5%	18 14%	94 72.9%	33 25.6%	2 1.6%

**Table 4:** Distribution of OH categories (G = good, M = moderate, P = poor) in pre- and post-brushing evaluation.

		OH POST BRUSHING																	
		TOTAL			Sextant									Surface					
					1			2			3			Buccal			Lingual		
		G	M	P	G	M	P	G	M	P	G	M	P	G	M	P	G	M	P
OH PRE BRUSHING	G	0	0	0	1	0	0	1	0	0	2	0	0	26	10	0	1	0	0
	M	39	24	1	40	23	1	47	18	1	48	31	1	15	67	11	74	24	0
	P	16	44	5	14	42	8	13	40	9	15	26	6	41	77	11	6	22	2

**Table 5:** Mean and standard deviation (SD) of PI pre- and post-brushing differences in the surfaces and locations evaluated. ^+^ *p* value. The U Mann–Whitney test was applied. * Statistically significant, *p* < 0.05.

PI Improvement	Mean ± SD	Gender	Hand Dominance	VMSRF	BAPS
		*p* Value ^+^	*p* Value	*p* Value	*p* Value
Total	0.88 ± 0.608	0.159	0.422	0.012 *	0.473
Sextant 1	0.85 ± 0.605	0.107	0.318	0.159	0.915
Sextant 2	0.86 ± 0.578	0.015 *	0.188	0.068	0.707
Sextant 3	0.79 ± 0.642	0.032 *	0.905	0.246	0.361
Buccal	0.95 ± 0.528	0.245	0.757	0.057	0.177
Lingual	0.84 ± 0.481	0.119	0.076	0.409	0.191

## Data Availability

The raw data presented in this study are available on request from the corresponding author due to privacy restrictions.
